# Adherence to Perioperative Antimicrobial Prophylaxis in Children in the Settings of Neurosurgery, Otorhinolaryngology, and Orthopedics

**DOI:** 10.3390/antibiotics14020125

**Published:** 2025-01-24

**Authors:** Dimitra Dimopoulou, Athina Tsakali, Maria M. Berikopoulou, Anastasia Dimopoulou, Vasiliki Kamposou, Dimitrios Panagopoulos, Christos-Sotiris Papadakis, Vasileios Tokis, Konstantina Pouli, Georgios Bozonelos, John Anastasopoulos, Konstantinos Antonis, Nektarios Papapetropoulos, Athanasios Michos

**Affiliations:** 1 2nd Department of Pediatrics, “Aghia Sofia” Children’s Hospital, 11527 Athens, Greece; dimi_med@hotmail.com (D.D.); athinatsakali@hotmail.com (A.T.); maria-berikopoulou@hotmail.com (M.M.B.); 2 Department of Pediatric Surgery, “Aghia Sofia” Children’s Hospital, 11527 Athens, Greece; natasa_dimo@hotmail.com; 3 Department of Otorhinolaryngology, “Aghia Sofia” Children’s Hospital, 11527 Athens, Greece; vickykabosou@gmail.com (V.K.); arispap@outlook.com (N.P.); 4 Department of Neurosurgery, “Aghia Sofia” Children’s Hospital, 11527 Athens, Greece; dimpanayop@gmail.com; 5 1st Department of Orthopedic Surgery, “Aghia Sofia” Children’s Hospital, 11527 Athens, Greece; hristos.papadakis91@gmail.com (C.-S.P.); vtokis96@yahoo.com (V.T.); gbozonelos@hotmail.com (G.B.); kantonis71@gmail.com (K.A.); 6 2nd Department of Orthopedic Surgery, “Aghia Sofia” Children’s Hospital, 11527 Athens, Greece; konstantinapouli@yahoo.gr (K.P.); jnanastasopoulos@gmail.com (J.A.); 7 Division of Infectious Diseases, 1st Department of Pediatrics, National and Kapodistrian University of Athens, 11527 Athens, Greece

**Keywords:** perioperative antimicrobial prophylaxis, children, guidelines, compliance, adherence, neurosurgery, orthopedics, otorhinolaryngology, surgical site infections

## Abstract

**Introduction**: Data about compliance with perioperative antimicrobial prophylaxis (PAP) guidelines in the pediatric population are limited. This study aims to evaluate PAP adherence in pediatric surgical subspecialty departments. **Methods**: A prospective cohort study was conducted from September 2023 to October 2024 at “Aghia Sophia” Children’s Hospital, Athens, Greece. Children <16 years old undergoing surgical procedures in the neurosurgery (NS), orthopedics (OP), and otolaryngology (ORL) departments were included. Data on demographics, surgical characteristics, and PAP practices (timing, agent, duration, and redosing) were collected and compliance with the international guidelines was evaluated. **Results**: A total of 301 children were included, with a median age (IQR) of 7 (8) years. PAP was received by 249/301 (82.7%) children (100% in the OP and NS, and 48% in the ORL). However, indications for PAP had 50.8% of children: 102/103 (99%) in the NS, 47/98 (47.9%) in the OP, and 4/48 (8.3%) in the ORL. Most children received broad-spectrum or combination of antimicrobials and/or antibiotics for longer duration. Appropriate PAP according to the guidelines was administered in 0% children in NS, 2% in OP, and 2.1% in ORL. Multivariable analysis in the ORL regarding the use of PAP revealed that shorter procedures (≤60 min; OR: 22.9, *p* = 0.003) and clean wounds (OR: 33.4, *p* < 0.001) were significantly associated with not using PAP. **Conclusions**: This study highlights gaps in the PAP guideline adherence in pediatric surgical departments, and the need for educational interventions to improve compliance and reduce antimicrobial use. Based on these findings, we plan to implement an educational intervention in order to optimize PAP practices in the pediatric population.

## 1. Introduction

Surgical Site Infections (SSIs) are defined as infections that develop within 30 days following surgery, or within one year in cases where an implant is involved. They are among the most common healthcare-associated infections (HAIs), contributing to extended postoperative hospitalizations, additional surgical interventions, higher morbidity and mortality, and healthcare costs [1,2]. More specifically, they represent up to 20% of all HAIs in the USA with an estimated annual cost of USD 3.3 billion. They also extend hospitalizations by approximately 9.7 days with an increased cost of more than USD 20,000 per admission [3,4,5].

Perioperative antimicrobial prophylaxis (PAP) plays a crucial role in reducing the rate of SSIs, along with other factors, such as adherence to basic infection-control strategies, the surgeon’s experience and technique, the duration of the procedure, hospital and operating-room environments, instrument sterilization issues, preoperative preparation, perioperative management, and the underlying medical condition of the patient, which may result in a reduction in SSI rates [6].

Ideally, an antimicrobial agent for surgical prophylaxis should prevent SSIs and especially, SSI-related morbidity and mortality, leading to a decrease in the hospital stay duration and healthcare-associated costs [7,8]. In addition, the optimal PAP should not cause any adverse effects or any adverse consequences for the microbial flora of the patient or the hospital environment [7,8]. Therefore, an antimicrobial agent should be active against the pathogens most likely to contaminate the surgical site and it should be administered in an appropriate dosage and timing that ensures adequate serum and tissue concentrations during the period of potential contamination [9,10,11]. Furthermore, PAP should have a good safety profile and should be administered for the shortest effective duration to limit adverse effects, resistance development, and costs [9,10,11]. So, the selection of appropriate PAP is based on cost, safety profile, ease of administration, pharmacokinetic profile, and bactericidal activity. Finally, antimicrobial agents with the narrowest spectrum of activity are recommended in order to be effective in preventing SSIs [12]. The appropriate use of PAP is essential to achieve optimal outcomes in surgical patients, as it can reduce the rate of SSIs by up to 50% [13]. According to the recommendations, PAP is indicated for clean-contaminated and contaminated surgeries, as well as clean procedures with a high risk of severe outcomes, such as surgeries involving prosthetic implants where even a rare infection can have serious consequences [12]. The current guidelines generally advocate the administration of a single intraoperative dose of PAP within 60 min before the surgical incision (or up to two hours for vancomycin and fluoroquinolones). Additional intraoperative dosing may be needed for prolonged surgeries, but routine antibiotic administration beyond 24 h is generally not recommended [4,14,15,16].

The appropriate use of PAP according to the guidelines is essential to attain optimal patient outcomes as inappropriate dose, timing, and drug choice can raise the risk of SSI, especially in vulnerable pediatric patients, leading to increased duration of hospitalization and overall healthcare costs [17,18]. Nonadherence to the current guidelines, including extensive use of broad-spectrum antimicrobials and the prolonged duration of PAP, could increase the risk of Clostridium difficile infections and contribute to the development of multidrug-resistant organisms, particularly vancomycin-resistant enterococci [19,20,21,22]. In addition, adhering to PAP guidelines ensures that pediatric patients receive the correct dosage, maximizing efficacy while minimizing potential side effects [23]. So, adherence to the PAP guidelines in children is a cost-effective and safe practice for improving health outcomes in this population.

Limited published data on adherence to the PAP guidelines in the pediatric population are available and adherence rates in different surgical departments are rarely documented separately [6,14,15]. To date, evidence about compliance with the PAP guidelines in the pediatric population in our area is limited. In response to the need for improved compliance with the PAP guidelines in other surgical departments, we designed a study to evaluate the current practices in the orthopedic, neurosurgery, and otolaryngology departments in a tertiary pediatric hospital.

## 2. Results

A total of 301 children (males: 59.5%) were included in the study with a median age (IQR) of 7 (8) years old. The demographic and surgical procedure characteristics of children are presented in Table 1. More specifically, 103 (34.2%) children were operated in the neurosurgery department, 98 (32.5%) in the orthopedic department, and 100 (33.2%) in the otolaryngology department. Twenty-two (7.3%) children were oncology patients [20 (19.4%) in neurosurgery, 1 (1%) in orthopedic, and 1 (1%) in otolaryngology), while only one patient was receiving immunosuppressive therapy. There was no child with underlying diabetes mellitus or neutropenia. The surgical wounds were characterized as clean (*n* = 257, 85.4%) or clean-contaminated (*n* = 44, 14.6%).

A total of 249/301 (82.7%) children received PAP (100% of children in the orthopedic and neurosurgery departments, and 48% in the otolaryngology department). However, indications for PAP had 50.8% of children (102/103 (99%) in the neurosurgery, 47/98 (47.9%) in the orthopedic and 4/48 (8.3%) in the otolaryngology department (Table 2).

Most children received broad-spectrum antibiotics or a combination of antimicrobial agents in contradiction to PAP guidelines and/or antibiotics for longer duration. The appropriate antibiotic regimen for PAP according to guidelines was administered to 12.3% of children (35.7% in the orthopedic, 4.2% in the otolaryngology, and 0% in the neurosurgery department). In the neurosurgery department, the most frequently used antimicrobials were Clindamycin (56/103, 54.4%), Cefuroxime (43/103, 41.7%), and Cefepime (43/103, 41.7%), followed by Teicoplanin (34/103, 33%), Ceftriaxone (15/103, 14.6%), Vancomycin (5/103, 4.8%), and Ceftazidime (1/103, 0.97%). The majority of the patients (93/103, 90.3%) received a combination of two antimicrobials. In the orthopedic department, the most common antimicrobials administered were Cefoxitin (46/98, 46.9%) and Ceforanide (32/98, 32.6%), followed by Amoxicillin/Clavulanate acid (13/98, 13.3%), Cefepime (8/93, 8.2%), Cefuroxime (6/98, 6.1%), Clindamycin (5/98, 5.1%), Teicoplanin (2/98, 2.04%), and Cefaclor (1/98, 1.02%). A combination of two antimicrobials was administered in 21.4% of the patients. In the otolaryngology department, the predominant antimicrobial prescribed was Amoxicillin/Clavulanate acid (42/48, 87.5%), followed by Ceforanide (7/48, 14.6%), Clindamycin (3/48, 6.3%), Amoxicillin (1/48, 2.1%), Ceftriaxone (1/48, 2.1%), and Cefprozil (1/48, 2.1%). A combination of two antimicrobials was given to 14.6% of the patients.

Concerning the duration of PAP, only 2.6% of the children (6% in the orthopedic, 4.2% in the otolaryngology, and 0% in the neurosurgery department) discontinued PAP within 24 h after surgery completion according to the instructions (Table 2). The median (IQR) duration of antimicrobial administration was 4 (2) days in neurosurgery, 4 (3) days in orthopedics, and 6 (2) days in the otolaryngology department.

Adherence according to the PAP guidelines on timing, agent selection, duration, and redosing, was observed in 0/103 (0%) of neurosurgery, 2/98 (2%) of orthopedics, and 1/48 (2.1%) of otolaryngology children (Table 2).

Regarding the otolaryngology department, as 52/100 (52%) of children undergoing surgery did not receive PAP, and 1/48 (2.1%) of the rest received appropriate PAP, the total compliance rate was 53%. For this reason, specifically for children undergoing otolaryngological procedures, a multivariable analysis was conducted to evaluate the factors associated with the administration or not of PAP according to the international guidelines. The analysis showed that children undergoing otolaryngology procedures were more likely not to receive PAP when the duration of the procedure was </= 60 min [OR (95%CI): 22.9 (2.88–182.2), *p* = 0.003] and the wound was clean compared to clean-contaminated [OR (95%CI): 33.4 (7.57–146.9), *p* < 0.001] (Table 3).

During the 6-month follow-up post-surgery, only one child was diagnosed with SSI in the neurosurgery department. This diagnosis was established through follow-up conducted via telephone communication, where their caregiver reported the infection and it was confirmed by the neurosurgeon. No case of SSI was reported in the orthopedic and otolaryngology departments.

## 3. Discussion

This study aimed to evaluate adherence to the PAP guidelines in pediatric patients in the neurosurgery, orthopedic, and otolaryngology departments in our area. In general, compliance with the PAP guidelines was suboptimal across all specialties, although the indication for PAP was high in most surgical procedures.

Broad-spectrum or combination of antibiotics and extended durations of administration were frequently observed, contradicting international recommendations. Especially, children were much more likely to receive PAP for longer than the appropriate duration.

Although studies on adherence to PAP in pediatric patients are limited, the compliance rates reported in this study are lower than those stated in the existing literature. In most studies, the adherence rate to PAP in children undergoing general surgery, including multiple specialties—Pediatric Surgery, Urology, Plastic Surgery, Cardiovascular Surgery, orthopedics, neurosurgery, and otorhinolaryngology—although the adherence rates for each specialty were not reported separately, ranges from 22% to 29% [13,24,25].

Data from studies conducted in the Department of General Pediatric Surgery at Children’s Hospital “Aghia Sofia” in Greece revealed a low adherence of 5.9–6.2% in terms of both the correct antimicrobial agent and duration, which was later improved to 77.1% after an educational intervention [26,27]. Another study showed that the overall adherence to guidelines was 39.6%, with variation between different domains and surgical subspecialties: high compliance with otorhinolaryngology (up to 80%) and moderate adherence to orthopedics and neurosurgery, ranging between 30% and 40% [28]. In Germany, a PAP audit in pediatric oncology patients undergoing neurosurgery showed that the correct antimicrobial type was administered in approximately 80%, while PAP was discontinued within 24 h in approximately 34–40% of the patients [29]. In adults undergoing neurosurgery procedures, the duration of the PAP was inappropriately prolonged in 82.8% of the procedures, but the choice of antimicrobial agents was optimal in 95% of the procedures [30].

The incidence of SSI during a six-month follow-up remained minimal (1%), with only one case reported in the neurosurgery department, as broad-spectrum antimicrobials were used for extended durations in contradiction to the international guidelines. These findings are consistent with other studies, which indicate that the SSI rate before educational intervention and management ranges from 1.0 to 2.7% [27,31,32,33]. More specifically, the SSI rate in pediatric patients undergoing general surgery was calculated to be 0.93% in the pre-intervention period and remained stable (0.92%) in the post-intervention period [27]. Other studies from Italy also confirmed these data, which identified that the rate of SSI was 2.5–2.7% before the intervention, while there was no significant difference in the SSI rate before and post-intervention [32,33]. Another retrospective study conducted on children undergoing general surgery in the United States demonstrated that the SSI rate was 2.37% [31]. Furthermore, this study showed that the children who received inappropriate PAP had a 1.7-fold increase in the risk of SSI compared to the children who received PAP within the recommended guidelines, and the children who received antibiotics were more likely to suffer an SSI compared to those who did not [31].

The multivariable analysis revealed that adherence in patients with otolaryngology was significantly associated with shorter procedures and clean wound classification. In a prospective study conducted on children who underwent surgery, including orthopedic, otolaryngology, and neurosurgery, the overall adherence to the guidelines regarding PAP was 22.1% and the rate of PAP administration was significantly lower, and the duration was shorter when the surgical procedure was clean [13]. Furthermore, the rate of PAP administration was higher when the duration of the procedure was longer [13]. The inappropriate antimicrobial agent and the longer PAP courses may be due to the surgeon’s anxiety and fear of wound infections or to past experiences of infectious complications. Therefore, a lack of awareness of the appropriate guidelines appears to be the main barrier to compliance with PAP guidelines, and educational intervention and/or antimicrobial stewardship is warranted to improve adherence rates [34].

The implementation of guidelines for the administration of appropriate PAP should be supervised by a well-trained physician with leadership skills [27,35,36,37]. This individual should be responsible for the adherence to guidelines for antimicrobial selection, ensure the appropriate duration of PAP is maintained, and encourage practice improvements by reviewing performance data and reinforcing the guidelines [27,35,36,37]. Once the guidelines have been disseminated and implemented and the changes in PAP practices have occurred, it is essential to conduct a comprehensive evaluation [27,35,36,37]. Ongoing audits of current practices along with feedback strategies are required to sustain the changes and further reinforce these improvements over time [27,35,36,37].

The limitations of the present study include that it represents data from a single pediatric hospital, which can limit the generalizability of the findings to other institutions or regions with different healthcare practices and resources. However, because the surgical procedures that were analyzed are commonly performed in children, our findings could help other hospitals improve the administration of PAP. In addition, the relatively small sample size, particularly when analyzing adherence within specific departments, may reduce statistical power and the ability to identify certain associations. Also, data collection based on the modified CDC Denominator for Procedure form and self-reported practices by surgeons may introduce reporting bias. PAP was administered without direct supervision, potentially leading to a lower compliance rate with the recommended guidelines. However, this is the first prospective study conducted across three different pediatric surgical specialties in the largest tertiary pediatric hospital in Greece, addressing an issue with limited international data available.

## 4. Materials and Methods

A prospective cohort study on surgical procedures was carried out in the departments of orthopedics, neurosurgery, and otolaryngology of “Aghia Sophia” Children’s Hospital in Athens, which is the largest pediatric hospital in Greece, serving approximately 40% of the pediatric population in the metropolitan area.

The study was conducted between September 2023 and October 2024. All pediatric patients, under 16 years of age, subjected to one or more surgical procedures, as defined by the National Healthcare Surveillance Safety Network (NHSN) and the Centers for Disease Control and Prevention (CDC), were eligible to be included in our study [38]. Patients who underwent procedures without full closure of the skin incision due to drains or other objects were excluded. Pediatric patients with a pre-existing infection at the time of surgery, who received antimicrobials for therapeutic purposes, were also excluded from the study.

A modified version of the CDC’s Procedure Denominator form was completed for each pediatric patient. After collection, the data were categorized into four major groups: (1) preoperative data, including demographics, underlying conditions, and anthropometric and perinatal characteristics; (2) perioperative data, including the type and route of antimicrobials administrated, the timing, and the duration of PAP; (3) surgical procedure details; and (4) postoperative details, covering therapeutic antibiotic use, length of postoperative hospital stay, and the incidence of SSI. The appropriateness of PAP was evaluated as the administration of preoperative doses of the correct antimicrobial agents for specific surgical procedures and the correct duration of AP for less than 24 h according to the recommendations of the American Society of Health System Pharmacists [12]. Each patient was monitored until discharge, followed by communication 30 days after surgery, or at six months in the case of implant placement, to evaluate possible SSIs.

According to the guidelines, PAP is indicated and may be beneficial in surgical procedures associated with a high rate of infection, such as clean-contaminated or contaminated procedures [12]. PAP is also recommended in specific clean procedures, involving prosthetic implants, where although infection is rare, it could result in severe consequences of infection. However, PAP is not indicated for the majority of clean surgical procedures [12]. On the other hand, PAP is generally justified for most clean-contaminated procedures. In contrast, the use of antimicrobials for dirty procedures or established infections is categorized as treatment for a presumed infection rather than prophylaxis [12].

The research protocol was approved by the Research Ethics Committee of the “Aghia Sophia” Children’s Hospital (protocol number: 8096/06.04.2023).

Statistical analysis involved descriptive statistics and inferential methods to assess associations between surgical characteristics and adherence to the PAP guidelines. Categorical variables were calculated using absolute counts (N) and percentages (%). The duration of PAP was expressed as median and interquartile range. Odds ratios (ORs) and 95% confidence intervals (CIs) were calculated to quantify the likelihood of appropriate PAP practices based on factors such as wound class, duration of the procedure, emergent status, implant use, and type of procedure. To address potential confounders, multivariate logistic regression was performed. This approach allowed simultaneous adjustment for multiple variables, isolating the independent effect of each characteristic on PAP compliance. Statistical significance was defined at 0.05. Statistical analysis was performed using the IBM SPSS Statistics software (version 29.0.2.0 Armonk, NY, USA: IBM Corp).

## 5. Conclusions

In conclusion, this study indicated gaps in the adherence to the PAP guidelines in pediatric surgical patients regarding the specialties of neurosurgery, orthopedic, and otolaryngology, with the overall compliance rates remaining low. Specifically, inappropriate use of broad-spectrum antibiotics and prolonged PAP durations were observed, potentially increasing the risk of antimicrobial resistance and other complications. The study findings emphasize the need for multidisciplinary educational intervention tailored to surgical teams, focusing on the rationale behind PAP guidelines, the consequences of nonadherence, and practical strategies for compliance. Based on the findings of this study regarding compliance rates in PAP, we plan to implement an educational intervention, which is an effective strategy to optimize clinical PAP practices in the pediatric population in terms of the appropriate choice of antibiotic agents and the appropriate duration of PAP and to reduce the unnecessary use of antibiotics and consequently, antimicrobial resistance.

## Figures and Tables

**Table 1:** Demographic and surgical procedure characteristics of the children (N = 301) hospitalized in the neurosurgery, orthopedic, and otolaryngology departments.

Characteristics	Neurosurgery (N = 103)	Orthopedics (N = 98)	ENT (N = 100)
**Sex**			
*Male*	62 (60.2%)	58 (59.1%)	59 (59%)
*Female*	41 (39.8%)	40 (40.8%)	41 (41%)
**Age (years) [Median (IQR)]**	4 (10)	12 (6)	6 (4)
**Type of Procedure**			
*V-P Shunt*	30 (29.1%)	-	-
*Cranioplasty*	15 (14.6%)	-	-
*Craniotomy-Structure Removal*	29 (28.1%)	-	-
*Others*	29 (28.1%)	-	-
*Osteosynthesis*	-	24(24.5%)	-
*ORIF*	-	7 (7.1%)	-
*Exostosis Removal*	-	6 (6.1%)	-
*Material removal*	-	11 (11.2%)	-
*Others*	-	50 (51%)	-
*Tonsillectomy*	-	-	9 (9%)
*Adenectomy*	-	-	12 (12%)
*Tonsillectomy and Adenectomy*	-	-	23 (23%)
*Mastoidectomy*	-	-	2 (2%)
*Others*	-	-	54 (54%)
**Wound Class**			
*Clean (C)*	101 (98.1%)	98 (100%)	58 (58%)
*Clean-contaminated (CC)*	2 (1.9%)	0 (0%)	42 (42%)
*Contaminated (CO)*	0 (0%)	0 (0%)	0 (0%)
*Dirty Infected (D)*	0 (0%)	0 (0%)	0 (0%)
**Endoscopic Procedure (Yes)**	6 (5.8%)	11 (11.2%)	4 (4%)
**Duration of Procedure** ≤**60 min**	103 (100%)	98 (100%)	100 (100%)
**Implant (Yes)**	16(15.5%)	45 (45.9%)	23 (23%)
**Emergent Procedure (Yes)**	57(55.3%)	40 (40.8%)	2 (2%)
**Duration of hospital stay (days) [Median (IQR)]**	6 (1)	4 (2)	1 (1)

ENT: Ear, Nose, and Throat; IQR: interquartile range; ORIF: Open reduction and Internal Fixation.

**Table 2:** Perioperative antibiotic prophylaxis practices in children (N = 301).

Characteristics	Neurosurgery (N = 103)	Orthopedics (N = 98)	ENT (N = 100)
**Received PAP**	103/103 (100%)	98/98 (100%)	48/100 (48%)
**Indication for PAP among those who received PAP**	102/103 (99%)	47/98 (47.9%)	4/48 (8.3%)
**PAP according to the international guidelines (timing, redosing, antimicrobial agent, and duration)**	0/103 (0%)	2/98 (2%)	1/48 (2.1%)
**Antimicrobial agent according to PAP guidelines**	0/103 (0%)	35/98 (35.7%)	2/48 (4.2%)
**Duration according to PAP guidelines**	0/103 (0%)	2/98 (2%)	1/48 (2.1%)
**Discontinued within 24 h**	0/103 (0%)	6/98 (6.1%)	2/48 (4.2%)
**Median (IQR) duration of PAP (days)**	4 (2)	4 (3)	6 (2)

ENT: Ear, Nose, and Throat; IQR: interquartile range; PAP: perioperative antibiotic prophylaxis.

**Table 3:** Compliance with appropriate perioperative antimicrobial prophylaxis (PAP) according to the international guidelines by characteristics in the otolaryngology department.

Characteristics	Odds Ratio	95% Cl	*p*-Value
**Age**	1.05	0.84–1.33	0.66
**Wound Class**			
*Clean (C)*	33.4	7.57–146.9	**<0.001**
**Duration of Procedure**			
*≤60 min*	22.9	2.88–182.2	**0.003**
**Implant (Yes)**	2.19	0.59–8.10	0.24
**Endoscopic Procedure (Yes)**	0.25	0.01–5.26	0.38
**Type of Procedure**			
*Adenectomy*	1.40	0.17–11.55	0.75
*Tonsillectomy and Adenectomy*	7.38	0.77–71.01	0.08
*Others*	1.66	0.30–9.12	0.56

CI: confidence interval. Bold numbers indicate that they are statistically significant (*p* < 0.05).

## Data Availability

The data underlying this article will be shared upon reasonable request to the corresponding author.
